# Sepsis Diagnostics: Intensive Care Scoring Systems Superior to MicroRNA Biomarker Testing

**DOI:** 10.3390/diagnostics10090701

**Published:** 2020-09-16

**Authors:** Fabian Link, Knut Krohn, Anna-Maria Burgdorff, Annett Christel, Julia Schumann

**Affiliations:** 1 Clinic for Anesthesiology and Surgical Intensive Care, University Hospital Halle (Saale), 06120 Halle, Germany; fabian.link2@student.uni-halle.de (F.L.); am.burgdorff@gmail.com (A.-M.B.); annett.christel@uk-halle.de (A.C.); 2 Core Unit DNA Technologies, Medical Faculty, Leipzig University, 04103 Leipzig, Germany; Knut.Krohn@medizin.uni-leipzig.de

**Keywords:** sepsis, inflammation, biomarker, sequential organ failure assessment (SOFA) score, miRNA, cytokines

## Abstract

Sepsis represents a serious medical problem accounting for numerous deaths of critically ill patients in intensive care units (ICUs). An early, sensitive, and specific diagnosis is considered a key element for improving the outcome of sepsis patients. In addition to classical laboratory markers, ICU scoring systems and serum miRNAs are discussed as potential sepsis biomarkers. In the present prospective observational study, the suitability of miRNAs in sepsis diagnosis was tested based on proper validated and normalized data (i.e., absolute quantification by means of Droplet Digital PCR (ddPCR)) in direct comparison to classical sepsis markers and ICU scores within the same patient cohort. Therefore, blood samples of septic intensive care patients (*n* = 12) taken at day of admission at ICU were compared to non-septic intensive care patients (*n* = 12) and a healthy control group (*n* = 12). Our analysis indicates that all tested biomarkers have only a moderate informative power and do not allow an unequivocal differentiation between septic and non-septic ICU patients. In conclusion, there is no standalone laboratory parameter that enables a reliable diagnosis of sepsis. miRNAs are not superior to classical parameters in this respect. It seems recommendable to measure multiple parameters and scores and to interpret them with regard to the clinical presentation.

## 1. Introduction

Sepsis represents a serious medical problem that affects about two percent of hospital admissions in developed countries [1] and remains one of the leading causes of death in critically ill patients in intensive care units (ICUs) [2]. To improve the outcome of septic patients, therapy has to start as soon as possible [3], which demands a quick and precise diagnosis. Consequently, the Surviving Sepsis Campaign declared the verification of rapid diagnostic tests as a sepsis research priority [4]. At present various biomarkers have been assessed for their suitability in sepsis diagnostics, while few could be established in clinical practice [5]. The most commonly used markers encompass white blood cell count (WBC) as well as the acute phase proteins C-reactive protein (CRP) and procalcitonin (PCT) [6]. A major disadvantage of these inflammation parameters is their limited specificity since they do not allow for an unambiguous distinction between septic and other critically ill patients [5,6,7]. Further inflammatory mediators involved in sepsis pathogenesis are the pro-inflammatory cytokines tumor necrosis factor-α (TNF-α), interferon-γ (IFN-γ), and the interleukins IL-1β and IL-6 [8,9]. Cytokine concentration in the serum of septic patients, though, was found to be very variable and strongly dependent on the time of blood collection which restricts the diagnostic usability [8,10,11,12,13,14,15,16]. Indeed, the only cytokine commonly used in clinical practice for sepsis diagnostics is IL-6 in neonatology [17]. For outcome prediction of septic patients in ICUs, various clinical parameters are combined into complex scores. The sequential organ failure assessment (SOFA) score and the simplified acute physiology score II (SAPS II) are well established with respect to a high prognostic significance [18,19]. However, while there are studies on the accuracy of outcome prediction, the suitability for initial diagnostics in sepsis has hardly been investigated. Recently, serum-circulating miRNAs were discussed as new sepsis biomarkers [20,21,22,23,24,25,26,27,28,29,30,31,32]. miRNAs are small, single-stranded, and non-coding nucleotides involved in the regulation of gene expression [33]. According to miRBase (release 22.1) there are 1917 precursors and 2654 mature miRNAs in humans [34]. miRNAs are present in all body fluids and can, therefore, be easily analyzed in a patient’s blood sample. In the medical field of oncology certain miRNAs have already been confirmed as biomarkers for specific tumor entities [35]. With regard to sepsis diagnostics, the situation is less clear. There are scientific publications proposing certain miRNAs as sepsis biomarkers [20,23,26,31,32], but study results are characterized by great discrepancies with respect to the recommended miRNAs. These conflicting data may be due to insufficient normalization. In fact, the majority of studies are characterized by a lack of validity as they merely use screening data or on qPCR data normalized using inappropriate housekeepers like U6 snRNA [36,37]. As a consequence, no miRNA marker has been established as a sepsis biomarker in clinical practice so far [38,39,40]. Against this background, the aim of the present study was to evaluate the suitability of miRNAs in sepsis diagnostics based on proper validated and normalized data in direct comparison to classical sepsis markers and ICU scores within the same patient cohort.

To avoid bias caused by improper normalization, miRNA quantification was performed by means of ddPCR. In this method, samples with a calibrated nucleic acid concentration are dispersed in droplets before polymerase chain reaction starts [41]. After thermocycling, the fluorescence signal is read for all droplets individually [41]. Data processing is based on the Poisson distribution to correct for variant allocation of nucleotides in droplets [41]. Previous studies have shown high accuracy and reliability of data for investigations on miRNAs [42,43]. Thus, the strength of this study is the sophisticated implementation of ddPCR, which allows for an absolute quantification of miRNAs thereby bypassing the critical issue of selecting an appropriate normalization model.

## 2. Materials and Methods

### 2.1. Ethics

This prospective observational study was performed at the University Hospital Halle (Saale, Germany). The study protocol was approved by the responsible Ethics Committee of the Medical Faculty of the Martin Luther University Halle-Wittenberg without any ethical objections to the conduct of the study (approval code: 2016-24, approval date: 20 April 2016). All experiments were performed in accordance with relevant guidelines and regulations. In consultation with the ethics committee, a group size of *n* = 12 was defined per study group in order to evaluate whether the concept of using miRNAs as sepsis biomarkers is likely to yield results. Only in case of a positive evaluation based on this number of included patients and volunteers the study protocol provided for a second step in terms of an expansion of the group size to *n* = 100 per group. All study participants or their next of kin gave written informed consent.

### 2.2. Study Population and Sample Collection

Three study groups were investigated: healthy control (HC) group, septic intensive care (SIC) group, and non-septic intensive care (NIC) group. The HC group represented blood donors matching the requirement profile of the hospital’s blood donation service. SIC and NIC groups were recruited at the ICU of the Clinic for Anesthesiology and Surgical Intensive Care, University Hospital Halle (Saale, Germany). Enrolment took place from August 2016 till February 2018. Exclusion criteria were age under 18 years, simultaneous or previous (within 30 days prior to study inclusion) participation in a clinical trial, lack of consent, pregnancy, AIDS, palliative situation, immunosuppressive therapy, active gastrointestinal bleeding, or the need for immediate surgery. Allocation to the SIC group was based on the Sepsis-3 criteria with evidence of severe infection and recurrent organ failure via clinical, radiological, and laboratory findings [44]. ICU patients with signs of recurrent organ failure and pathological leucocyte count or CRP values not fulfilling Sepsis-3 criteria were assigned to the NIC group. The determination of the laboratory parameters and the classification in the corresponding reference range was performed by the central laboratory of the University Hospital.

Blood samples were taken at day of admission using serum separating tubes (S-Monovette, Sarstedt, Nümbrecht, Germany). According to the study protocol, blood sampling for subsequent miRNA analysis was permitted within the first 24 h after admission to ICU concerning both the standardized procedures of initial sampling in intensive care patients for diagnostic purposes and time dependent variations of miRNA expressions. Blood samples were centrifuged immediately at 1400× *g* for 15 min. Serum was taken off and stored at −80 °C.

Clinical and epidemiological parameters of the SIC and NIC patients (age, gender, main diagnosis, routine blood test results, results of blood culture, SOFA score, SAPS II, and 28-day mortality) were collected in case reports.

### 2.3. Literature Research

Electronic searches were performed on 1 March 2018 in the following databases: (i) PubMed/MEDLINE, and (ii) Web of Science. Search terms included “sepsis”, “miRNA*”, “biomarker*” combined with the Boolean operator “AND”. An abstract screening was performed to identify eligible articles based on following exclusion criteria: no original research article, no human model, participant’s age < 18 years, reference to sepsis-related secondary diseases, no qPCR validated data. Remaining articles were assessed in full text regarding miRNA-based sepsis biomarkers.

### 2.4. RNA Isolation

Total RNA was isolated using TriFast FL (VWR/Peqlab, Erlangen, Germany) following the manufacturer’s standard protocol for serum extraction. To purify samples, a routine precipitation of RNA was performed based on Ambion^®^ molecular biology grade 5 M ammonium acetate solution (Invitrogen/Thermo Fischer Scientific, Dreieich, Germany). Concentration and quality of the isolated RNA was determined using the NanoDrop spectrophotometer (Thermo Fischer Scientific, Dreieich, Germany). An absorbance quotient A260/280 > 1.8 was considered appropriate for further procedures.

### 2.5. Deep Sequencing and Analysis of Deep Sequencing Data

miRNA expression was analyzed by next generation sequencing (NGS) in the Core Unit DNA, Medical Faculty, Leipzig University by means of an Illumina HiScan sequencer (Illumina Inc., San Diego, CA, USA) as previously described [45]. Samples were processed using the TruSeq Small RNA Prep kit v2 (Illumina Inc., San Diego, CA, USA) following the manufacturer’s standard protocol. Size restriction (140–165 bp), purification, and quantification of barcoded libraries were performed using the library quantification kit, Illumina/Universal (KAPA Biosystems, Woburn, MA, USA). For cluster generation up to 10 libraries per lane were factored using an Illumina cBot. An Illumina HighScanSQ sequencer was used to perform 50 bp sequencing based on version 3 chemistry and flowcell following the manufacturer’s standard protocol.

For deep sequencing data analysis, the adapter sequences were removed from raw sequences using Cutadapt software, version 1.9.1. To verify that small RNAs other than mature miRNAs are filtered out from the data only sequences 15–27 bases long were analyzed. These reads were aligned to the human genome (GRCh38: NCBI_Assembly:GCA_000001405.15) as well as mature sequences of miRBase v21 using the bowtie aligner. An error rate of 1 nt per mature miRNA sequence was accepted. For data compression to bam format, Samtools were used. Mapped reads count was determined using the R/Bioconductor programming environment by application of the ShortRead library. Sequence analysis after mapping against mature sequences of miRBase v21 is not affected by counts for other small RNAs (e.g., rRNA, tRNA fragments) because these sequences are not present in the reference sequences. For mapping against the whole genome reference rRNA or tRNA fragments are usually below 1–2% in the 15–27 bp fraction. Reads that map to their respective loci were removed before analysis using genome coordinates. Normalization of data was performed by independent application of the DESeq 2 and the TMM (EdgeR) algorithm. NGS was performed for four individuals of every study group.

### 2.6. Droplet Digital PCR

Complementary DNA (cDNA) was synthesized using the miRCURY LNA RT Kit (QIAGEN, Hilden, Germany) according to standard protocol. To avoid bias due to incorrect normalization, expression analysis of the human miRNAs miR-26b-5p, miR-122-5p, miR-143-3p, miR-146a-5p, miR-193-3p, miR-223-3p, miR-486-3p, and miR-486-5p was performed by means of the housekeeper-independent Droplet Digital PCR technology (BioRad, Munich, Germany) following the manufacturer´s instructions. Suitable miRCURY LNA miRNA PCR assay primers (QIAGEN, Hilden, Germany) and ddPCR EvaGreen Supermix (Bio-Rad, Munich, Germany) were used. PCR reaction was performed in a T100 Thermal Cycler (Bio-Rad, Munich, Germany). Measurement of positive droplets per µL sample was performed on a QX200 ddPCR Droplet Reader (Bio-Rad, Munich, Germany). Based on the droplet count and according to Poisson Distribution, absolute nucleic acid copy count was calculated. Data output was converted into nucleic acid copy count per ng RNA. ddPCR reaction was performed in duplicates for each individual analyzed.

### 2.7. ELISA

Quantification of the cytokines IL-1β, IL-6, TNF-α, and IFN-γ in the serum was performed using suitable human ELISA kits (Invitrogen/Thermo Fischer Scientific, Dreieich, Germany) following the manufacturer´s standard protocol. Absorbance was determined on an EnSpire multimode plate reader (PerkinElmer, Rodgau, Germany) at 450 nm wavelength. ELISA was performed in duplicates for each individual analyzed.

### 2.8. Statistical Analysis

If not stated otherwise data are shown as median ± interquartile range (IQR). To identify significant differences, a nonparametric Mann–Whitney U test was performed in two group comparisons and a nonparametric Kruskal–Wallis one-way analysis of variances by ranks followed by Dunn’s corrected multiple testing was used in three group comparisons. Categorical variables were compared using the χ^2^ test. The statistical analysis was carried out by means of the program GraphPad Prism 6 (GaphPad Software, La Jolla, CA, USA). In all cases, *p* < 0.05 was assumed to indicate significant differences.

## 3. Results

### 3.1. Characteristics of the Study Population

This study included 36 study participants enrolled in three groups: healthy control (HC) group (*n* = 12), septic intensive care (SIC) group (*n* = 12), and non-septic intensive care (NIC) group (*n* = 12). Epidemiological, clinical, and routine laboratory parameters of the study population are shown in Table 1. Overall median age was 58 (IQR 73–47). While SIC and NIC group were quite comparable in age (*p* = 0.322), the HC group was significantly younger (*p* < 0.01). This was to be expected due to the requirement profile of the blood donation service. The gender distribution was quite balanced (*p* = 0.66) between all groups. Also, there were no significant differences between SIC and NIC group regarding routine laboratory parameters (hemoglobin, hematocrit, erythrocyte or thrombocyte cell count, creatinine, bilirubin). A major difference, however, was seen regarding the 28-day mortality rate. In comparison to the NIC group, the number of deaths in the SIC group was twice as high (31% versus 17%). Of note, for the vast majority of SIC patients (11 out of 12 enrolled patients) blood cultures were positive, which underscores the validity of data collected. The remaining patient had clinical evidence of infection with extensive endocarditis. Sepsis was mainly caused by urogenital infection (42%), followed by intestinal perforation (25%), and endocarditis (17%).

### 3.2. Classical Sepsis Markers and Scores

**Figure 1:**
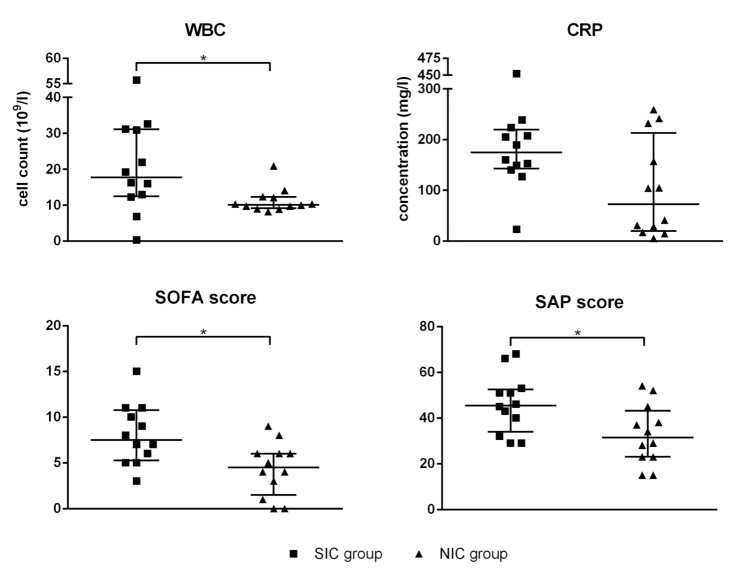
White blood cell count (WBC), serum level of C-reactive protein (CRP), the sequential organ failure assessment (SOFA) score, and the simplified acute physiology (SAP) score II were determined for every patient enrolled in the septic intensive care (SIC) and the non-septic intensive care (NIC) group. The blood sample was taken immediately after admission to the intensive care unit (ICU). Data are presented as median ± interquartile rande (IQR; *n* = 12); the asterisk denotes a significant (*p* < 0.05) group difference.

ICU patients were routinely tested for WBC as well as serum levels of CRP at day of admission. In addition, the SOFA score and the SAPS II were determined immediately after hospitalization in intensive care. Compared to the NIC group, WBC was significantly elevated in SIC patients (1.9-fold), while CRP showed a non-significant trend (Figure 1). Significant differences between the SIC group and the NIC group were also found with respect to the established sepsis scores SOFA and SAPS II (Figure 1). Compared to the NIC group, SIC patients were characterized by increased levels of both the SOFA score (1.9-fold) and the SAPS II(1.4-fold).

### 3.3. Cytokines

**Figure 2:**
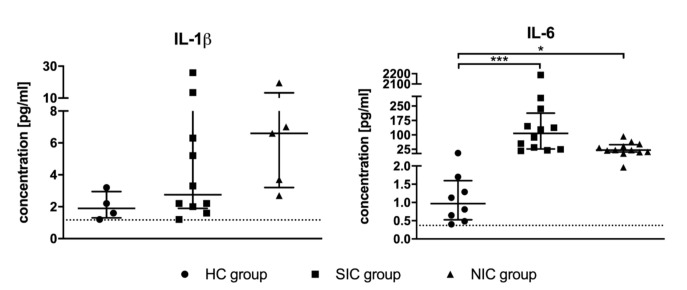
Serum concentrations of the pro-inflammatory cytokines interleukin 1β (IL-1β) and interleukin 6 (IL-6) were determined by means of suitable ELISA kits. The blood sample was taken immediately after admission to the intensive care unit (ICU). Data are presented as median ± IQR; the asterisk denotes a significant group difference (* *p* < 0.05, *** *p* < 0.001).

Serum levels of the pro-inflammatory cytokines TNF-α, IFN-γ, IL-1β, and IL-6 were measured using highly sensitive ELISA assays. Remarkably, the concentrations of TNF-α and IFN-γ were found to be below the assay’s detection limit for the majority of tested individuals independent of group assignment (TNF-α detection limit of 0.5 pg/mL exceeded by only 11 of 36 study participants; IFN-γ detection limit of 0.24 pg/mL exceeded by only 15 of 36 study participants). The concentration of IL-1β could be determined for 19 of 36 study participants, the statistical analysis showed no significant differences between groups (Figure 2). IL-6 could be measured for all test subjects except for four healthy test persons (Figure 2). Compared to the HC group, the serum level of IL-6 was significantly increased in both SIC and NIC patients. However, the group comparison between SIC and NIC patients revealed no significant differences, which questions the suitability of these parameter as specific sepsis marker.

### 3.4. miRNAs

#### 3.4.1. Screening

**Figure 3:**
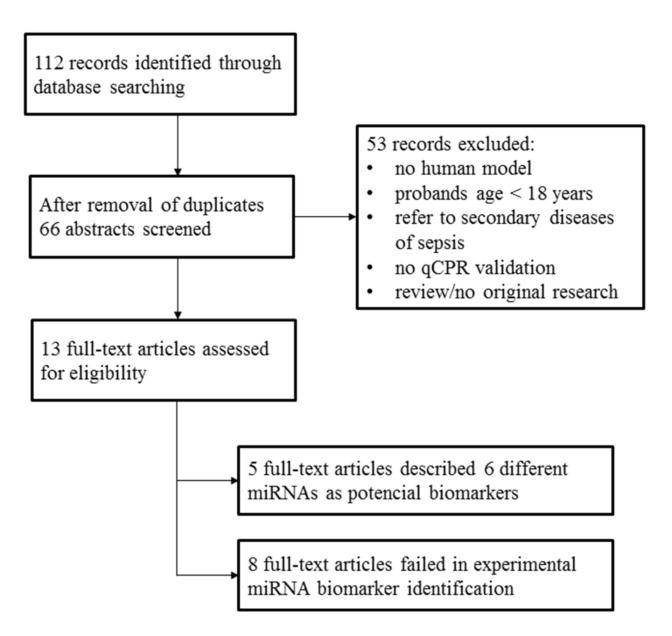
Literature research was performed on 1 March 2018 utilizing the databases PubMed/MEDLINE and Web of Science by means of the search terms “sepsis”, “miRNA*”, “biomarker*” combined with the Boolean operator “AND”.

Two independent screening approaches were used to identify potential miRNA-based sepsis markers. First, a structured web search for scientific publications dealing with this topic was performed. Details of the literature search are shown in Figure 3. Second, an NGS (next generation sequencing) profiling of serum miRNAs was conducted based on four representative individuals of each study group.

The literature search resulted in 13 full-text original research articles; eight of these described a failed experimental validation of a miRNA-based sepsis marker. Nevertheless, we identified five full-text articles that proposed particular miRNAs as potential sepsis markers (Table 2). These studies were characterized by a remarkable lack of agreement on the suggested miRNA-based markers. In summary, the following six miRNAs were suggested: miR-26b-5p [32], miR-122-5p [23], miR-143-3p [31], miR-146a-5p [20,26], miR-193-3p [26], and miR-223-3p [20,26].

NGS analysis resulted in the detection of 256 miRNAs with a great variability in abundancy. Selection of potential miRNA-based sepsis markers was linked to the following criteria: (i) high abundancy (>100 reads in average); and (ii) effect strength of Cohen’s D > 0.8, which is considered a large effect size [46]. The only miRNAs fulfilling both criteria were miR-486-3p and miR-486-5p.

#### 3.4.2. Validation

To validate the potential miRNA-based sepsis markers identified in the previous screening process a housekeeping gene independent absolute quantification by means of ddPCR was performed. All eight miRNAs identified during screening were included into the validation process.

Intra-group validity was assessed by means of the coefficient of variation (CV) for every miRNA investigated (please see Supplementary Materials Table S1). All miRNAs showed a high variability within the healthy control group, complicating the specification of a threshold value. Copy counts also fluctuated strongly in the SIC group and the NIC group, which suggests that there may be further interfering factors.

**Figure 4:**
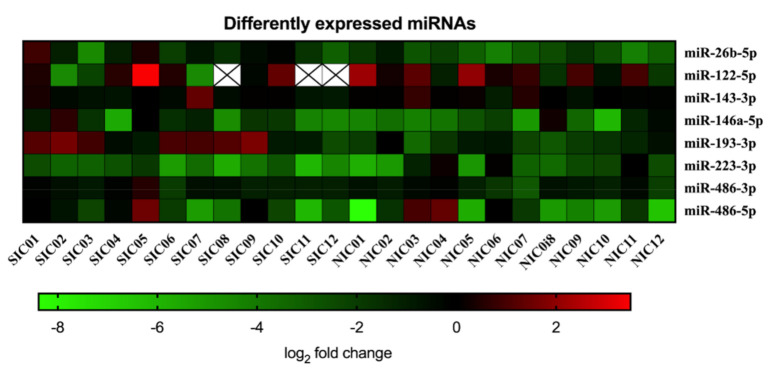
Serum copy counts of miRNAs miR-26b-5p, miR-122-5p, miR-143-3p, miR-146a-5p, miR-193-3p, miR-223-3p, miR-486-3p, and miR-486-5p were determined in septic intensive care patients (SIC) and non-septic intensive care patients (NIC) by means of the Droplet Digital PCR technology. Data are presented for each individual patient as log_2_ fold change of miRNA compared to the healthy control group.

For estimation of the influence of sepsis on the particular miRNA, expression changes were calculated for each individual SIC and NIC patient as fold changes compared to the HC group and were visualized in a scaled heat map (Figure 4).

**Figure 5:**
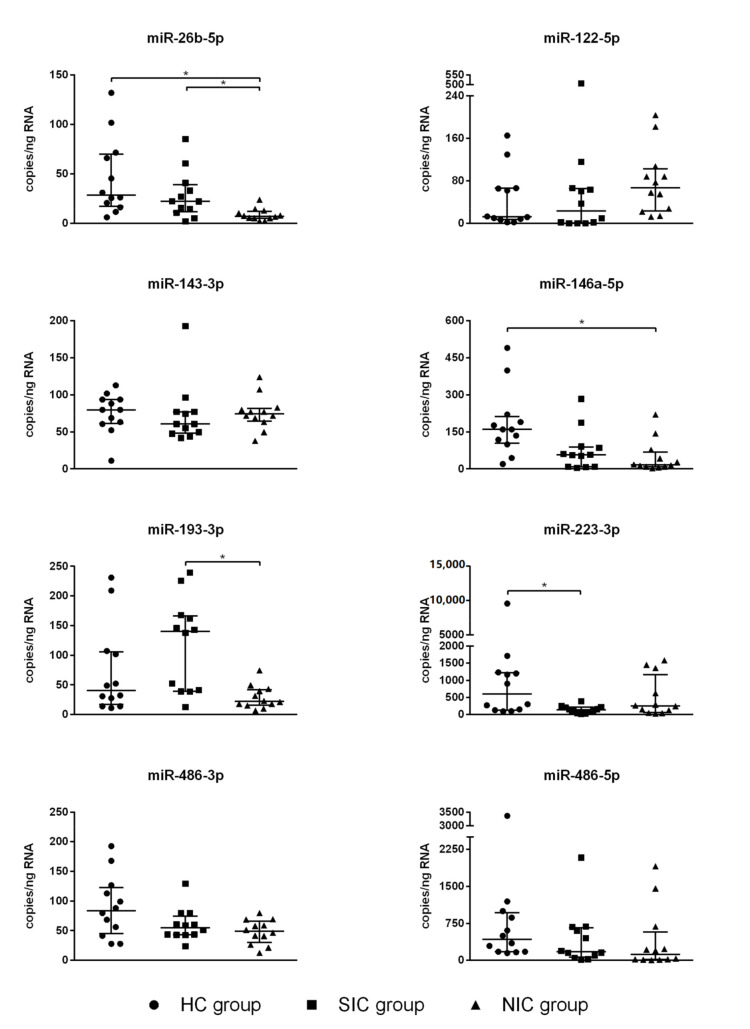
Serum copy counts of the miRNAs miR-26b-5p, miR-122-5p, miR-143-3p, miR-146a-5p, miR-193-3p, miR-223-3p, miR-486-3p, and miR-486-5p were determined by means of the Droplet Digital PCR technology. The blood sample was taken immediately after admission to the intensive care unit (ICU). Data are presented as median ± IQR (*n* = 12, *n* = 2); the asterisk denotes a significant (*p* < 0.05) group difference.

The resulting picture does not show clustering of miRNA expression levels, which prevents a correct classification of patients as septic or non-septic on this basis. Comparing the SIC group, NIC group, and HC group, four miRNAs (miR-122-5p, miR-143-3p, miR-486-3p, miR-486-5p) showed no significant group differences at all (Figure 5). For miR-146a-5p, a significant group difference was found between the HC group and the NIC group (Figure 5). Expression of miR-223-3p was found to be diminished in SIC patients compared to the HC group (Figure 5). However, the group comparison between SIC and NIC patients revealed almost equal expression levels of both miR-146a-5p and miR-223-3p, which makes these miRNAs unsuitable for the identification of sepsis patients on ICUs (Figure 5). The only miRNAs with demonstrably significant differences in expression between SIC and NIC patients were miR-26b-5p and miR-193-3p (*p* < 0.05; Figure 5). Nevertheless, suitability of these miRNAs as sepsis markers is limited by the fact that there was no clear distinction between healthy and critically ill patients (either septic or non-septic; Figure 5).

### 3.5. Diagnostic Capability

**Figure 6:**
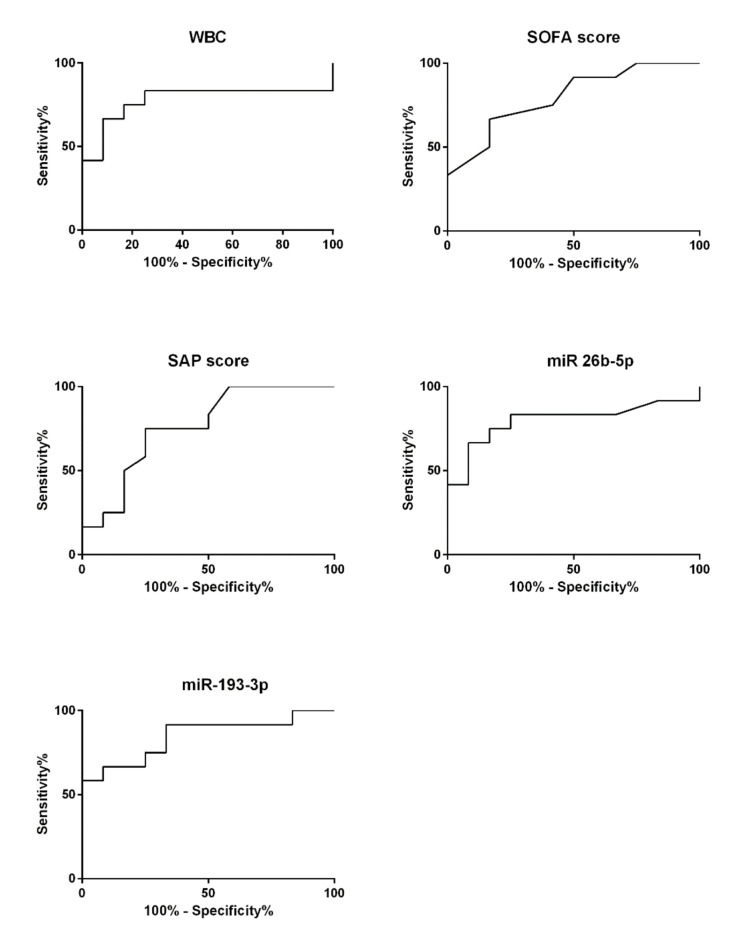
Receiver operating characteristic (ROC) curve analysis was performed on all parameters that showed significant group differences between the septic intensive care (SIC; *n* = 12, *n* = 2) and the non-septic intensive care (NIC; *n* = 12, *n* = 2) groups.

In order to estimate the diagnostic capability of the tested sepsis markers that enabled a statistically valid differentiation between SIC and NIC patients (i.e., WBC, SOFA score, SAPS II, miR-26b-5p, miR-193-3p) a ROC curve analysis was performed (Figure 6). The calculated AUC for WBC was 0.78 ± 0.11 (95% CI: 0.56–0.99; *p* = 0.02). The sepsis scores, which have so far been used primarily for prognostic purposes, reached comparable values of 0.80 ± 0.09 (95% CI: 0.62–0.98; *p* = 0.01; SOFA score) and 0.76 ± 0.10 (95% CI: 0.56–0.96; *p* = 0.03; SAP score II). The miRNA miR-26b-5p showed a similar diagnostic capability reaching an AUC of 0.80 ± 0.10 (95% CI: 0.60–0.99; *p* = 0.01). A slightly better suitability to differentiate between the SIC and NIC groups was indicated for miR-193-3p reaching an AUC of 0.85 ± 0.08 (95% CI: 0.69–1.00; *p* = 0.004).

To test whether the integration of miRNA quantity as an additional parameter could optimize diagnostic informative power, we modified the calculation of the SOFA score. Based on the mean and standard deviation of miR-193-3p copy counts in the NIC group, we added one score point for a number of copies exceeding two standard deviations and up to four points for each further standard deviation above. Direct comparison of the SOFA score and modified SOFA score is displayed in supplementary Figure S1. The AUC for the modified SOFA score was 0.91 ± 0.06 (95% CI: 0.80–1.00; *p* = 0.0007), which is a relevant improvement compared to the unmodified scoring system.

## 4. Discussion

A key element for improving the outcome of sepsis patients—who despite all developments in intensive care medicine continue to show a high mortality rate—is the identification of biomarkers that allow an early, sensitive, and specific diagnosis [44]. A particular challenge is the differentiation from other patients in critical care who suffer, for instance, from burn injuries or polytrauma. So far, numerous studies have tested various parameters, such as acute phase proteins, cytokines, and different scoring systems [47,48]. However, no universally accepted standard in clinical practice could be established with regard to the ambiguous study situation [4]. The classical laboratory parameters including WBC, CRP, and cytokines are widely used in sepsis diagnostics. Advantages are broad availability and relatively low costs of determination [6]. Yet it is known that many other factors besides sepsis have an influence on the serum concentration of these markers, e.g., hepatic function, immunosuppression, antibiotic therapy, or time of blood sampling [5,6]. The low specificity of the classical laboratory parameters is also reflected by the data obtained in our study. WBC, CRP, and cytokine levels of the study patients were on average higher than the reference values for the general population, but a significant difference between septic and non-septic intensive care patients was observed only for WBC. The AUC of elevated WBC (0.78 ± 0.11) indicates an acceptable diagnostic accuracy. There are, however, sepsis courses with normal WBC or leukopenia that are not covered by this parameter [49].

With regard to the proven suitability of miRNAs as biomarkers in oncology, the determination of these nucleic acids is discussed as a potential tool for sepsis diagnostics as well. Our structured web search of the current scientific literature shows that the majority of relevant studies failed to identify a miRNA-based biomarker that allows a clear distinction between septic and non-septic intensive care patients. Besides, the comparison of the few proposed biomarker miRNAs reveals inconsistent study results on the basis of which no conclusive assessment can be made. The contradictory findings may possibly be explained by the normalization methods used for the qPCR-based miRNA quantification. It should be noted that many authors used qPCR housekeeping oligonucleotides, such as the snRNA U6, which are unsuitable under septic conditions and, therefore, lead to data sets with limited validity and reproducibility [36,37]. Against this background, we have re-examined all miRNA-based biomarkers that have been suggested in the scientific literature allowing for the first time a direct comparison of these miRNAs in the same study cohort. The list of the investigated miRNA-based biomarker candidates was extended by those miRNAs that could be identified in an experimental approach using NGS. To avoid distortions due to incorrect normalization, miRNA quantification was done using the housekeeper-independent method ddPCR, which has not been applied in this field of research until now. The ddPCR allows an absolute quantification of individual nucleotide molecules leading to an excellent quality and comparability of data. We could already confirm the validity of this method in an earlier publication on quantification of nucleic acids under septic conditions [45]. Accordingly, we considered it particularly appropriate for the objectives of this study. In our study none of the examined miRNAs, neither the candidates derived from literature nor the candidates derived from sequencing, showed a diagnostic power superior to other parameters. Of the eight miRNAs tested, two miRNAs (miR-26b-5p and miR-193-3p) displayed a significant difference between septic and non-septic intensive care patients at time of admission to ICU. However, it should be noted that these miRNAs did not allow the differentiation between healthy volunteers and septic patients. The diagnostic validity of these miRNAs is therefore questionable. One reason for this could be the rapidity with which miRNAs are up- or down-regulated under inflammatory conditions. According to an examination on mice there are considerable fluctuations within a few hours [50]. This considerably reduces the time window for blood sampling and thus the usability of miRNA-based sepsis biomarkers in clinical diagnostics. In fact, there might be significant changes in miRNA serum levels of particular miRNAs at certain points of sepsis progression. However, this is not relevant for the use of biomarkers, since such a parameter needs to be stable over time for a reliable diagnosis in clinical practice. Moreover, for three of the candidates (miR-122-5p, miR-143-3p, miR-486-3p) it has already been demonstrated that these are significantly influenced by gender and age [51]. Since it is unclear which other factors can influence the serum concentrations of miRNAs in sepsis patients, the eligibility of miRNAs in sepsis diagnostics appears doubtful.

The SOFA score and SAPS II achieved the most coherent results in this study. These scoring systems were developed in the 1990s to predict the outcome of patients in intensive care units independent of the underlying disease [52,53,54]. According to our data, both scoring systems allow the distinction of septic and non-septic intensive care patients with a relatively low variance and a high diagnostic quality (AUC of SOFA score: 0.80 ± 0.09; AUC of SAP score II: 0.76 ± 0.10), which may be explained by the complex composition of the scores that corresponds to the pathophysiology of sepsis as a multi-organ failure. Our data also indicate that the SOFA score can be further improved by including additional parameters. The miRNA miR-193-3p might be a possible item here in an updated version. For this reason, we recommend the use of complex scoring systems, especially the SOFA score, not only for prognostic but also for diagnostic purposes.

## 5. Conclusions

Summarizing, all markers described for sepsis have only a moderate informative power on their own and do not allow an unequivocal differentiation between septic and non-septic intensive care patients. Our study shows that the SOFA score is most likely to provide a reliable diagnosis but cannot provide certainty as a standalone. Hence, various parameters should be measured and always be considered in the context of the clinical picture and dynamics. A significantly increased SOFA value can, however, be a clear indication of the development of sepsis, especially if there is a clinically plausible suspicion of infection.

## Figures and Tables

**Table 1:** Epidemiological, clinical, and routine laboratory parameters of the study participants at admission.

Characteristic	HC Group	SIC Group	NIC Group
	*n* = 12	*n* = 12	*n* = 12
Age (years)	44.1 ± 19.5	61.5 (73–45)	72 (77.5–55.5)
Sex, male (%)	60	66	50
28-day mortality (%)	-	33	16.7
BMI	-	22.9 (26.5–21.0)	24.1 (28.7–23.1)
Hemoglobin (mmol/L)	-	5.9 (6.2–5.3)	6.4 (6.9–5.2)
Hematocrit (%)	-	27.0 (28.8–27.0)	0.28 (0.33–0.24)
Erythrocytes (10^12^/L)	-	3.2 (3.6–3.0)	3.2 (3.8–2.8)
Leukocytes (10^9^/L)	-	17.7 (31.2–12.5)	10.1 (12.3–9.1)
Thrombocytes (10^9^/L)	-	134.5 (307.0–100.0)	200.5 (262.0–142.5)
Creatinine (µmol/L)	-	101.5 (274–61)	101 (141–61)
Bilirubin (µmol/L)	-	12.2 (29.6–12.2)	8.5 (15.8–6.9)
SOFA score	-	7.5 (10.8–5.3)	4.5 (6.0–1.5)
SAPS II score	-	45.5 (51.5–38.0)	31.5 (39.8–23.0)

Data are presented as median (IQR); the asterisk denotes significance (*p* < 0.05) versus the HC group. BMI, body mass index; HC, healthy control; IQR, interquartile range; NIC, non-septic intensive care; SAPS II, simplified acute physiology score II; SIC, septic intensive care; SOFA, sequential organ failure assessment.

**Table 2:** miRNAs proposed in literature as sepsis markers.

Proposed Biomarker	Housekeeper Used	Article DOI
miR-26b-5p	Mean of hsa-miR-185-5p and hsa-miR-25-3p	10.1111/jcmm.13162
miR-122-5p	U6	10.1097/TA.0b013e31825a7560
miR-143-3p	U6	10.1177/0300060516645003
miR-146a-5p	Spike-in (mmu-miR-295)	10.1016/j.bbrc.2010.02.145
	U6	10.3892/etm.2013.937
miR-193-3p	U6	10.1097/TA.0b013e31825a7560
miR-223-3p	Spike-in (mmu-miR-295)	10.1016/j.bbrc.2010.02.145
	U6	10.1097/TA.0b013e31825a7560

## Supplementary Materials

The following are available online at https://www.mdpi.com/2075-4418/10/9/701/s1, Figure S1: title, Table S1: title, Video S1: title.
