# Supplementary material for: Sepsis Diagnostics: Intensive Care Scoring Systems Superior to MicroRNA Biomarker Testing
Source: Diagnostics (Basel). 2020 Sep 16;10(9):701. doi: 10.3390/diagnostics10090701 (PMC7555112; doi:10.3390/diagnostics10090701)
Supplement: Supplementary file 1 [file diagnostics-10-00701-s001.pdf]

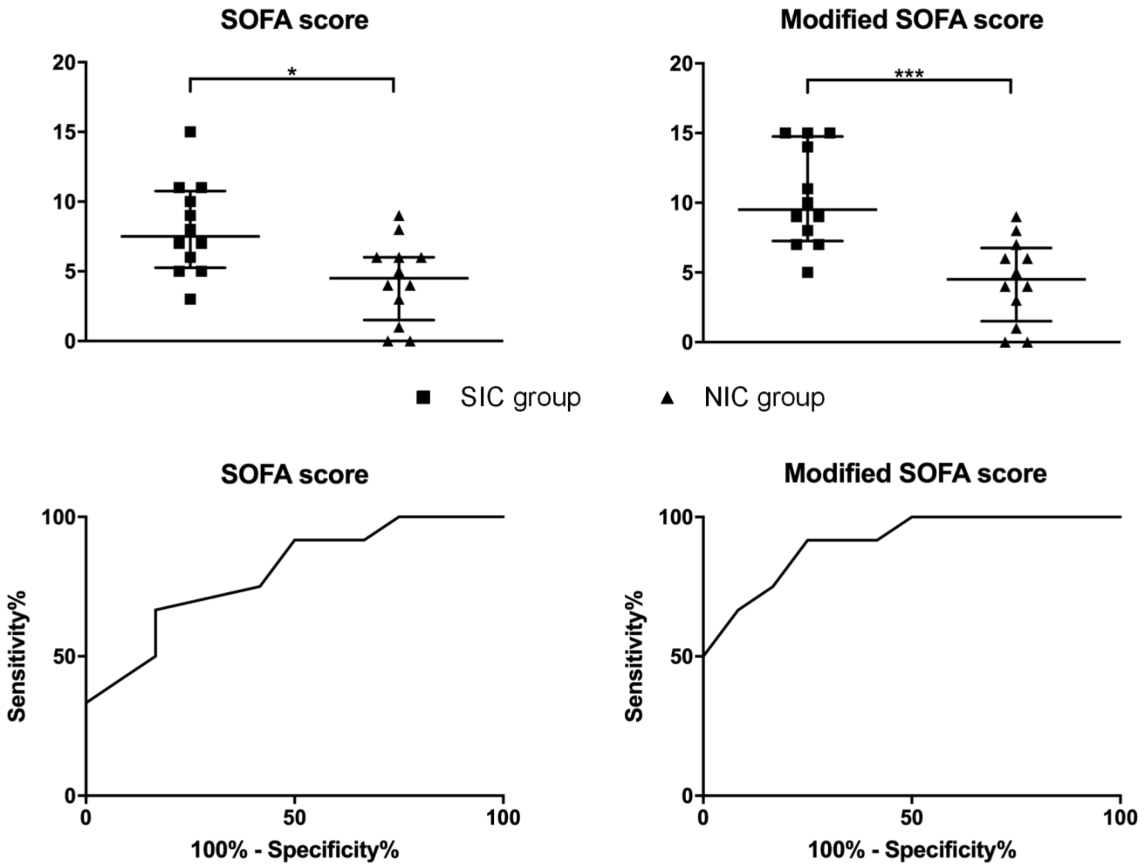

Supplementary Figure 1. The sequential organ failure assessment (SOFA) score was determined for every patient enrolled in the septic intensive care (SIC) and the nonseptic intensive care (NIC) group. Modification of SOFA score was based on copy count of the potential miRNA marker miR-193-3p. For determination of the modified SOFA score the mean and standard deviation of miR-193-3p copy counts in the NIC group were calculated. One score point was added for a number of copies exceeding two standard deviations and up to four points for each further standard deviation above. Data are presented as median ± IQR; the asterisk denotes a significant group difference (*$p < 0.05$, ***$p < 0.001$).

Supplementary Table 1. Intra-group variability of miRNA expression

**miRNA**	**HC group n = 12**	**SIC group n = 12**	**NIC group n= 12**
miR-26b-5p	84,9%	85,6%	69,5%
miR-92-3p	160,6%	93,5%	93,6%
miR-122-5p	120,7%	197,3%	79,9%
miR-143-3p	35,9%	56,2%	30,1%
miR-146a-5p	73,7%	110,7%	139,0%
miR-193-3p	103,2%	66,4%	67,0%
miR-223-3p	188,0%	74,4%	115,2%
miR-486-3p	57,9%	45,5%	43,0%
miR-486-5p	122,6%	134,5%	159,1%

To assess the intra-group variability of selected miRNAs, the coefficient of variation (CV) was calculated individually for the study groups. Data are presented as percentage.
